# Modern Homœopathy

**Published:** 1868-08

**Authors:** 


ARTICLE XXIX.

### MODERN HOMŒOPATHY.

COMMUNICATED.

From the *London Monthly Homœopathic Review*, for June, 1868, we learn that Lord Ebury gave vent to a feeling of regret that the Report of the London Homœopathic Hospital did not contain evidence of a greater development of the objects of the Institution. The number of patients was not very large, and the clinical lectures had been given up, "owing to the attendance being so scanty as greatly to discourage the lecturers."

We also find that the fourth object of the founders of the Hospital was, not is, "to let inquirers see of what homœopathic treatment consists, and *wherein it differs from other methods of cure.*" But the position is now assumed, that "when a physician adopts homœopathy he does not bind himself to use medicines on no other principle. His first and chiefest obligation is to the patient." His second duty only is due to homœopathy, for he must do the best within his knowledge for the promotion of his patients' recovery or relief. "When a case, or part of a case, is without the sphere of the homœopathic law; in other cases where his knowledge of the practice fails him; and, again, where a suitable palliative appears to be demanded, he is bound to use other than homœopathic means. This is always required in private practice," and is carried out in the London Homœopathic Hospital, although "a plan of treatment partly homœopathic and partly something else; one, in which one patient is treated in accordance with the law of similars, and another only partially so"—will fail in securing no less than four out of the five objects, to achieve which the Hospital was founded, *viz.:* to obtain statistics by which the success of homœopathic treatment *may* be compared with other methods of cure; to demonstrate the success of pure homœopathic treatment; to teach the system in all its purity, etc., etc. All these great objects are subservient to the interests of the sick, for the editor says:—

"We all know and admit that there are cases in which the most conscientious and painstaking (homœopathic) practitioners feel compelled to fall back upon auxiliary (allopathic) aids to eke out a modicum of relief, or indeed to assist in curing."

Accordingly, in the Londom Homœopathic Hospital, in which the cases "are not of so serious a character as one is accustomed to see under treatment in hospital," black wash is applied to syphilitic sores, and $\frac{1}{2}$-grain doses of mercury given internally. Cases of glandular enlargement in the neck were treated with tincture of iodine, painted on externally. For cases of continued sleeplessness $\frac{1}{48}$ of a grain of acetate of morphia was given every 15 minutes.

The Hospital physicians repudiated, one and all, the fallacies and absurdities of Hahnemann about infinitesimal doses and dilutions. They quietly told an inquiring physician "that the doses were not a part of *their* system; that like cures like was all *their* creed;" although it is not a matter of general or partial belief that morphia keeps people awake; that iodine produces enlargement of the glands; and black wash produces syphilis.

These Hospital physicians, and many others, have at last returned to the use of the medicines of the rational school in appreciable quantities; and the editor says the treatment in the above cases "was far too like allopathy to be advisable in a homœopathic hospital," although, "so long as the doses of mercury did not induce mercurialism, our opponents cannot (or rather should not) complain." He also says, he would avoid painting a glandular enlargement in the neck with tincture of iodine publicly in a homœopathic hospital, although he might unhesitatingly do it in private practice. And formally admits: "As regards the giving of morphia for sleeplessness, however necessary it may have been considered by the medical attendant, it cannot be called homœopathic treatment."

The homœopathic editor trusts his readers will thoroughly understand that he has no wish to object to those methods of treatment *per se*, although unsuitable for a hospital called homœopathic.

We are hardly surprised to find that Dr. Yeldham, the principal physician of the London Homœopathic Hospital, in what is called an excellent speech, delivered at the annual meeting of the Institution, referred to the possible expediency of a middle school, which would embrace both homœopathy and allopathy; and Dr. Reith said he knew many homœopathists who are prepared for the coalition.

The editor of the *Homœopathic Review* "has not the slightest doubt that some such step (or surrender) would be fraught with incalculable advantage to practical medicine," and it remains for us to see in what it should consist. If Hahnemann was so utterly mistaken about the efficacy of his infinitesimal doses; if every observation which he made upon the sick was an error; if every supposed cure was simply a recovery, is it not only not possible, but probable, that much of his reasoning and theorizing about the law *similia similibus curantur* is also false? If the frank and practical homœopathists must make such large concessions about the dose, will they not also be soon obliged to make equally large concessions about the theory? If we can judge from the following prescriptions, some of the homœopathists can make every required concession:

Quin. sulph. gr. viij; ext. cannibas ind. gr. iv; ext. aconit. *rad.* gr. iv. Make 8 pills; 1 every 3 or 4 hours. F——r.

Hydrarg. sub. mur. gr. iij; mass hydrarg. gr. iij; aloes gr. iij; podophyllin gr. $\frac{1}{8}$. Make 4 pills; to be taken at once. F.

Potassæ sulphuret ʒj; aq. ℥iv. Solve. M——d.

Pulv. alum erstæ ʒiiss; tannin ʒiiss; tinct. myrrhæ ℥j. For a gargle. M——d.

Magnesiæ sulphat. ℥iij; tinct. cort. aurant. ℥xvj; acid. sulph. aromat. ℥ss. M.

Ferri persulphat. ʒj; adipis. ℥j. M.

The *Medical Gazette* says: "Hahnemann taught that one grain of sulphur well rubbed up with 100 grains of sugar of milk could be developed into a medicine of tremendous energy. Some of his disciples of the Billy Barlow kind have made great improvements upon the simple proceeding of Hahnemann, and found out that 2 grains of sulphur mixed with 126 grains of conium, quinine, and morphine, are still more efficacious. See following prescription: Sulphur pura, gr. ij; ext. conii mac., dr. iss, or 90 grains; quiniæ sulph., sc. iss, or 30 grains; morph. sulph., gr. iij; podophyllin, gr. iij; *i.e.* 126 grains of conium, quinine, morphine, and podophyllin, to 2 grains of sulphur. Make 30 pills, each containing 3 grains of conium, 1 grain of quinine, $\frac{1}{10}$ grain of morphine, $\frac{1}{10}$ podophyllin, and $\frac{1}{15}$ grain of very pure sulphur, and give one or two several times a-day in all cases in which sulphur was formerly considered useful, namely, in dyspepsia, biliousness, psore, eruptions, constipation, etc."

